# Identification and analysis of YELLOW protein family genes in the silkworm, *Bombyx mori*

**DOI:** 10.1186/1471-2164-7-195

**Published:** 2006-08-03

**Authors:** Ai-Hua Xia, Qing-Xiang Zhou, Lin-Lin Yu, Wei-Guo Li, Yong-Zhu Yi, Yao-Zhou Zhang, Zhi-Fang Zhang

**Affiliations:** 1 The Sericultural Research Institute, Chinese Academy of Agricultural Sciences, Zhenjiang City, Jiangsu Province, 212018, China; 2 Institute of Biochemistry, Zhejiang Sci-Tech University, Hangzhou, Zhejiang Province, 310018, China; 3 The Biotechnology Research Institute, National Engineering of crop germplasm and genetic improvement, Chinese Academy of Agricultural Sciences, Beijing, 100081, China

## Abstract

**Background:**

The major royal jelly proteins/yellow (MRJP/YELLOW) family possesses several physiological and chemical functions in the development of *Apis mellifera *and *Drosophila melanogaster*. Each protein of the family has a conserved domain named MRJP. However, there is no report of MRJP/YELLOW family proteins in the Lepidoptera.

**Results:**

Using the YELLOW protein sequence in *Drosophila melanogaster *to BLAST silkworm EST database, we found a gene family composed of seven members with a conserved MRJP domain each and named it YELLOW protein family of *Bombyx mori*. We completed the cDNA sequences with RACE method. The protein of each member possesses a MRJP domain and a putative cleavable signal peptide consisting of a hydrophobic sequence. In view of genetic evolution, the whole *Bm *YELLOW protein family composes a monophyletic group, which is distinctly separate from *Drosophila melanogaster *and *Apis mellifera*. We then showed the tissue expression profiles of *Bm *YELLOW protein family genes by RT-PCR.

**Conclusion:**

A *Bombyx mori *YELLOW protein family is found to be composed of at least seven members. The low homogeneity and unique pattern of gene expression by each member among the family ensure us to prophesy that the members of *Bm *YELLOW protein family would play some important physiological functions in silkworm development.

## Background

Major royal jelly proteins and YELLOW proteins in Insecta, together with an orphan protein found in *Deinococcus radiodurans*, a radiation tolerant bacterium, form a protein family named the MRJP/YELLOW family [1,2]. Major royal jelly proteins (MRJPs) are initially identified as the major content of royal jelly (RJ) proteins, constituting 80%–90% of the total RJ proteins which play a central role in the honeybee development [3]. A recent report indicates that MRJP/YELLOW protein family in *Apis mellifera *includes at least 8 MRJPs (named MRJP1-8) and two homologues of the *Drosophila *YELLOW proteins, *Am*-YELLOW and *Am*-YELLOW-f [2]. Most MRJPs have the characteristic that there are repetitive segments encoding long homopetptides at the carboxyl terminal. The structure is thought of as the accessible form of storing nutrition [4]. *Apis mellifera *MRJP/YELLOW proteins may have higher physiological functions because at least one of the members expresses in the brain's mushroom body of the honeybee [5]. At the N-terminal of MRJP/YELLOW protein, there is a strong hydrophobic sequence functioning as putative signal peptide [6]. It should be noted that the term MRJP was created before knowing their physico-chemical properties. Later it was established that these proteins have physico-chemical properties similar to those of ovalbumin (storage egg-white protein) or serum albumin (major protein of serum) which are typical albunoid proteins and therefore researchers have proposed to rename major royal jelly proteins as apalbumins. Thus, apalbumin-1 will be designated as MRJP1, apalbumin-2 as MRJP2, and so on. This new terminology of honeybee larval diet proteins corresponds with reality that these proteins are presented not only in royal jelly, but also in worker and drone jellies [7].

The *Drosophila yellow *gene is related to normal larval and adult pigmentation and movement, and the mating behavior of male and female [8-10]. It encodes a simple transcription unit of two exons, encoding a 541 aa protein. Further researches indicate several novel *Drosophila *genes possessing a high identical MRJP conserved domain are termed as the *yellow *family [1]. With the achievement of the *Drosophila melanogaster *genome-sequencing project [11], the *Drosophila melanogaster yellow *gene family has grown to a total of more than 14 genes [12]. The *Drosophila melanogaster yellow-y *and *ebony *genes together determine the degree of melanization and its pattern [13]. The *yellow-f *and *yellow-f2 *genes have dopachrome-conversion enzyme activity that likely playing an important role during melanin biosynthesis in *Drosophila melanogaster *larvae, pupae and adults [14]. Furthermore, MRJP-like protein was also found and identified in the blood-sucking insect, possessing an agglutinin activity and probably intermediating in the evolution from *yellow*-like function towards royal jelly components [15].

To date, no related protein has been found in the non-insect metazoans except the orphan protein in the red pigmented bacterium *D. radiodurans *with 59% similarity to the *Drosophila *Yellow protein [16]. However, there is no report of a MRJP/YELLOW family protein in the Lepidoptera. With the declaration of the completion of EST library [17,18] and the achievement of genome sequence draft project [19,20] in *Bombyx mori*, the silkworm, as a model organism has been attracting more scientists. We searched the silkworm EST library by the BLAST method and found eight partial MRJP/YELLOW family genes in *Bombyx mori*. Using the SMART™ RACE Amplification method we completed seven of the cDNA sequences. The nucleotide acid and amino acid structures of the genes were analyzed, and the tissue expression profiles and phylogenetic analysis were also studied.

## Results

### Identification of *Bm *YELLOW protein family in the EST library

We obtained 74 ESTs of different length from the silkworm EST library, using the conserved domain in Yellow protein of *Drosophila melanogaster *to BLAST. We grouped and combined these sequences using the programs of DNASTAR and CLUSTAL X software packages. Seven *Bm *YELLOW protein family genes (GenBank: DQ358079–DQ358085) (Table 1) each contained a MRJP conserved domain were gained.

### Sequence analysis in *Bm *YELLOW protein family

#### Bm-yellow-d

The biggest group consisted of 21 ESTs derived from midgut, wing discs, ovary, fat body and pheromone gland. The assembled contig contained 1,510 nucleotide acids with incomplete 3'-terminal. Primers (Table 2) were designed depending on the assembled sequence, and 3'-RACE was performed using midgut cDNA as the template. Then the obtained 3'-RACE product was combined with the assembled contig and named *Bm-yellow-d*. The sequence of *Bm-yellow-d *was 1,678 bp long, containing an open reading frame of 1,341 bp encoding a protein 446 amino acids long. The stop codon was located 159 bp upstream of the poly (A) tail and no usual polyadenylation (AAUAAA) signal was found. The first 24 amino acids of the protein made a putative signal peptide and the location between amino acid residue 122 and residue 412 was the MRJP conserved domain.

The sequence of the *Bm-yellow-d *cDNA was almost completely covered by the sequence (GenBank: AADK01006220, AADK01006404, AADK01009017) readings from shotgun sequencing of the silkworm genome. This enabled us to identify the 6 introns in the genomic locus of *Bm-yellow-d*. Considering the incompleteness of the fifth intron, two primers (5'-gtttccaacgtgggaagactta, 5'-cgagaaacgtcgatactgtgtt) for PCR were designed basing on known sequences (GenBank: AADK01006404, AADK01009017), to complete the genomic sequence of *Bm-yellow-d*. Surprisingly, we got several amplification products of different sizes and the sequencing results showed that there might be an extensively high repeat area.

#### Bm-yellow-fa

Fifteen ESTs from pheromone gland, fat body or embryo were combined in an assembled contig 1,274 bp long absent 3'-terminal. After combined with the 3'-RACE product from pheromone gland, a 1,543 bp cDNA was gained and named *Bm-yellow-fa*. *Bm-yellow-fa *contained an open reading frame of 1,380 bp encoding a protein 459 amino acids long. The stop codon was located 164 bp upstream of the poly (A) tail, 31 bp upstream of which was a consensus polyadenylation signal (AAUAAA). A putative signal peptide was found in the first 17 amino acids of the protein. The complete MRJP conserved region stretched from residues 168 to 456.

Shotgun sequence readings of the silkworm Genome (GenBank: BAAB01099246, BAAB01003180, BAAB01177891, AADK01017861, BAAB01046234, AADK01031417, BAAB01168441, BAAB01050009, AADK01029979) could cover the whole of the *Bm-yellow-fa *cDNA. That enabled us to deduce that the genomic locus of *Bm-yellow-fa *contained 12 introns.

#### Bm-yellow-c

*Bm-yellow-c *was combined by the assembled contig 1,274 bp long, consisting of 10 ESTs from pheromone gland, ovary and fat body, and the 3'-terminal fragment obtained by 3'-RACE from pheromone gland. The gene was composed of 1,916 bp and contained an open reading frame of 1,224 bp, encoding a protein 407 amino acids long with a putative signal peptide in the first 18 amino acids and a MRJP conserved region in 118–406 amino acid residues. The stop codon was located 633 bp upstream of the poly (A) tail. No typical polyadenylation (AAUAAA) signal was found.

Shotgun sequence readings of the silkworm Genome (GenBank: BAAB01124731, AADK01025556, BAAB01064927, AADK01021354, BAAB01083903, AADK01026184) covered partial of the *Bm-yellow-c *cDNA, which enabled us to conclude at least 9 introns were contained in the genomic locus of *Bm-yellow-c*.

#### Bm-yellow-fb

A 941 bp long assembled contig consisted of 3 ESTs from pheromone gland, fat body or embryo. We obtained the 3'-terminal fragment by 3'-RACE from ovary and combined as the *Bm-yellow-c *gene 1,516 bp long. The gene contained an open reading frame of 1,257 bp, encoding a protein 418 amino acids long, with a putative signal peptide in the first 20 amino acids and a MRJP conserved region spanning 132–416 amino acid residues. The stop codon was located 188 bp upstream of the poly (A) tail, 14 bp upstream which was the consensus polyadenylation signal (AAUAAA).

Shotgun sequence reading of the silkworm Genome (GenBank: BAAB01155212, AADK01014564, AADK01037111, AADK01031942) covered all the *Bm-yellow-fb *cDNA. That enabled us to conclude that the genomic locus of *Bm-yellow-fb *contained 8 introns.

#### Bm-yellow-b

The EST from pheromone gland (GenBank: BP183406) 656 bp long fell in the smallest contig. We got a long 3'-RACE product from pheromone gland and assembled it with the EST sequence, and obtained the 2,045 bp *Bm-yellow-b *gene. The gene contained an open reading frame of 1,374 bp, encoding a protein 457 amino acids long with a putative signal peptide in the first 26 amino acids and a MRJP conserved domain spanning 135–413 amino acid residues. The stop codon was located 640 bp upstream of the poly (A) tail, and a consensus polyadenylation signal (AAUAAA) was located only 12 bp upstream of the tail.

Shotgun sequence readings of the silkworm Genome (GenBank: BAAB01152328, AADK01044238) covered the whole cDNA of *Bm-yellow-b *and no intron was identified in the genomic locus of *Bm-yellow-b*.

#### Bm-yellow-f2

Two ESTs from ovary and brain composed an assembled contig 731 bp long. The 3'-terminal fragment was obtained by 3'-RACE from ovary and combined with the former assembled contig, composing the cDNA of *Bm-yellow-f2*, which was 1,130 bp long. *Bm-yellow-f2 *cDNA contained an open reading frame of 855 bp, encoding a protein 284 amino acids long with a putative signal peptide in the first 17 amino acids and an incomplete MRJP conserved region spanning 125–216 amino acid residues. The stop codon was located 259 bp upstream of the poly (A) tail, while no consensus polyadenylation signal (AAUAAA) was found.

Shotgun sequence readings of the silkworm Genome (GenBank: AADK01003428, AADK01027742) covered partial of the cDNA of *Bm-yellow-f2*. That enabled us to conclude that at least 5 introns were contained in the genomic locus of *Bm-yellow-f2*.

#### Bm-yellow

A 1,045 bp assembled contig consisted of 10 ESTs from compound eye, pheromone gland, ovary and fat body was named *Bm-yellow*. With the result of 3'-RACE, we got a cDNA of 1,947 bp contained a complete open reading frame, encoding a protein of 514 amino acids. A putative signal peptide was found in the first 18 amino acids and a MRJP conserved domain was located from 115 to 399 amino acid residues. The stop codon was located 373 bp upstream of the poly (A) tail, and a consensus polyadenylation signal (AAUAAA) was found located 17 bp upstream of the poly (A) tail.

Shotgun sequence readings of the silkworm Genome (GenBank: BAAB01161029, AADK01014604) covered partial of the *Bm-yellow *cDNA. It showed that no less than 3 introns were in the genomic locus of *Bm-yellow*.

### Alignment and phylogeny of *Bm *YELLOW protein family proteins

Homology alignment was performed between the YELLOW protein families of *Drosophila melanogaster *and *Bombyx mori *at amino acid sequence level. The result showed that *Bm*-YELLOW-*d *shared the highest identity with YELLOW-*d2 *among *Drosophila melanogaster *YELLOWfamily (38.8%). *Bm*-YELLOW-*fa *and *Bm*-YELLOW-*fb *both shared the highest identity with YELLOW-*f *(42.9% and 35.1%, respectively), while *Bm*-YELLOW-*f2 *shared with YELLOW-*f2 *(21.1%). *Bm*-YELLOW-*c *shared the highest identity with YELLOW-*c *(50.5%), and *Bm*-YELLOW-*b *was the most similar to YELLOW-*b *with an identity of 23%. *Bm*-YELLOW was mostly homologous to *Drosophila melanogaster *YELLOW (51.2%).

**Figure 1:**
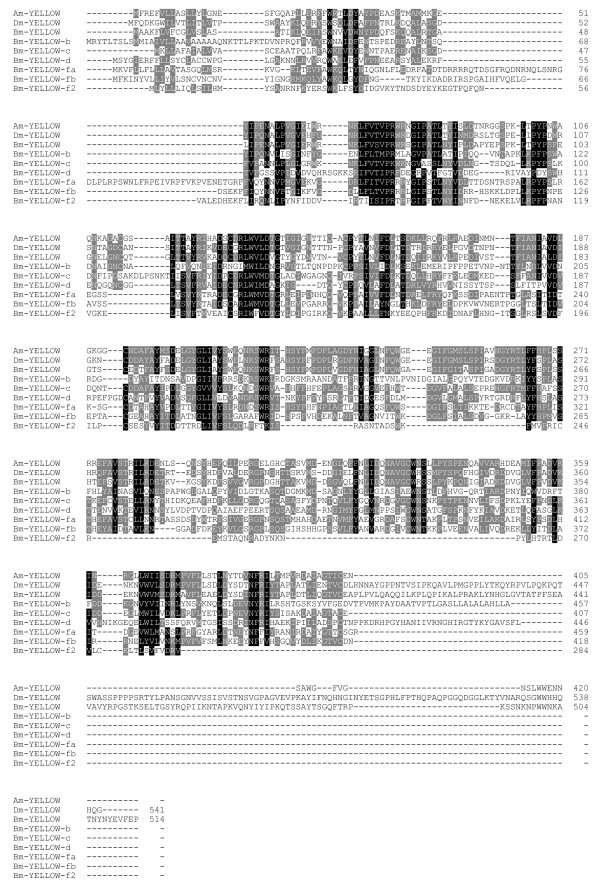
**Alignment of *Bm*-YELLOW proteins with that of *A. mellifera *and *D. melanogaster***. The protein sequences were aligned using CLUSTAL X and visualized with Genedoc software. Amino acids that are similar in 90% of aligned sequences are shaded black, 40%–60% grey.

The protein sequences of the *Bm *YELLOW protein family, with *Dm*-YELLOW and *Am*-YELLOW, were aligned using the CLUSTAL X software package and modified by GeneDoc software (Figure 1). The homology existed in the MRJP conserved domain of all the *Bm*-YELLOW family genes, but its similarity was lower than that of the *Apis mellifera *MRJP/YELLOW protein family and the *Drosophila melanogaster *YELLOW protein family. For example, though *Bm*-YELLOW-*fa *and *Bm*-YELLOW-*fb *shared the highest identity, it was only 41.3%. Surprisingly, some unique amino acid sequences were found in the members of the *Bm *YELLOW protein family such as residues 53 to 106 in *Bm*-YELLOW-*fa*, 26 to 33 in *Bm*-YELLOW-*b *and the C-terminal of *Bm*-YELLOW-*d *and *Bm*-YELLOW-*b*. The above information indicated that complicated divergence existed in the *Bm *YELLOW protein family.

**Figure 2:**
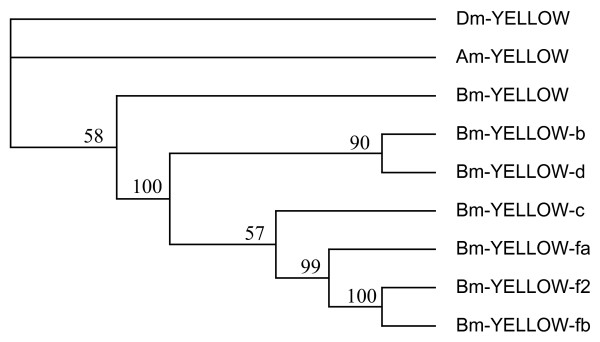
**Phylogeny of YELLOW family proteins of *B. mori***. The numbers forks indicate the number of times the group consisting of the species which are to the right of that fork occurred among the trees, out of 100 trees.

The phylogenetic tree was calculated from the aligned protein sequences using the neighbor-joining (NJ) method (Figure 2). The *Bombyx mori *YELLOW protein family, *Apis mellifera *YELLOW and *Drosophila *YELLOW formed three monophyletic groups independently. In the *Bm *YELLOW protein family group, *Bm*-YELLOW underwent the earliest divergence within the family.*Bm*-YELLOW-*c*, *Bm*-YELLOW-*fa*, *Bm*-YELLOW-*fb *and *Bm*-YELLOW-*f2 *might be classified into one paraphyletic sub-group. The other paraphyletic sub-group included *Bm*-YELLOW-*d *and *Bm*-YELLOW-*b*. The protein distance analysis showed that there was moderation support for an earliest divergence of *Bm*-YELLOW among the known members. The results of phylogenetic analyses showed that the *Bm *YELLOW protein family genes clearly formed a monophyletic group distant from *Apis mellifera *and *Drosophila melanogaster*.

### Tissue expression patterns of *Bm *YELLOW protein family

**Figure 3:**
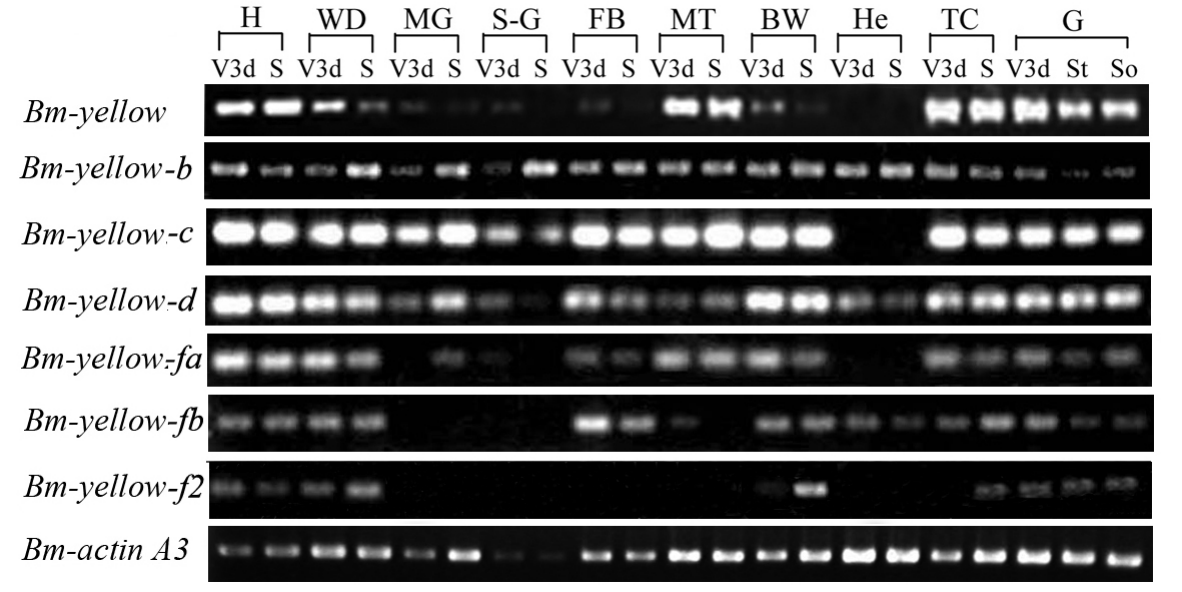
**Tissue expression profiles of the *yellow *family genes of silkworm**. RT-PCR of tissue templates including H = head, WD = wing discs, MG = midgut, SG = silk glands, FB = fat body, MT = malpighian tubules, BW = body wall, He = hemocyte, TC = trachea cluster, G = gonad, T = testis, O = ovary. V3d = 3^rd ^days of the 5^th ^instar larvae. S = 8^th ^days of the 5^th ^instar larvae (the beginning of spinning).

The RT-PCR was done to analyze tissue specific expression patterns of the *Bm *YELLOW protein family genes on the 3^rd ^and the 8^th ^days (matured silkworm) of the 5^th ^instar larvae (Figure 3). The tissues included head, wing discs, midgut, silk glands, fat body, malpighian tubules, body wall, hemocyte, trachea, and gonads (testis and ovary). *Bm-yellow-d *and *Bm-yellow-b *were transcripted in all the tissues, therein a low expression was observed of *Bm-yellow-d *in silk glands and *Bm-yellow-b *in the testis of matured silkworm.*Bm-yellow-fa *was obviously found not expressed in hemocyte. Notably high levels of *Bm-yellow-c *transcripts were readily detectable in all tissues except hemocyte. *Bm-yellow-fb *was not found in malpighian tubules, midgut and silk glands but found in others. *Bm-yellow-f2 *was only expressed in head, wing discs, body wall, pheromone gland and trachea. *Bm-yellow*, which was most similar to *Dm-yellow*, was expressed in most of the organs studied in the experiment. But it was hardly detectable in malpighian tubules, silk glands, fat body and body wall. We would note that even all of the numbers of *Bm-yellow *family had high expression level in gonads.

## Discussion

We obtained seven genes coding proteins each contains a conserved MRJP domain from the present silkworm EST library and named the silkworm YELLOW protein family genes. Each protein in the family possessed a putative cleavable signal peptide composed of a hydrophobic sequence, 17 to 26 residues long at the N-terminal, and a MRJP conserved domain. In the SilkDB [21,22], seven genes predicted from the silkworm genome draft sequence, each possess a putative MRJP conserved domain have been named as Bmb004831, Bmb006279, Bmb006279, Bmb010554, Bmb026342, Bmb030806, and Bmb033954. However, our results are different from theirs.

For the confidence of *Bm *YELLOW protein family members, we designed primer1 and primer2 based on the EST sequences that included 5' non-coding region except *Bm-yellow-d*. In order to validate the assemblage of *Bm-yellow-d*, we designed two primers containing complete coding region (5'-atgtcgtacggaatcgagcgat-3' and 5'-ctacagaaatgacacagcgcc-3'), and successfully cloned the entire open reading frame from the midgut cDNA. The length of *Bm-yellow-f2 *gene was the shortest and its MRJP conserved domain was truncated. But the search of its genomic sequence revealed that contig027742 (GenBank: AADK01027742) covered all the sequence of the last exon (at the nucleotide acid site from 747 to 1,140). All the above results proved the credibility of the seven cloned *Bm *YELLOW protein genes.

The *Apis mellifera *MRJP/YELLOW family and the *Drosophila melanogaster *YELLOW family resulted from a series of events of a duplication and subsequent divergence [6]. What analysis of cDNA sequence and genomic structure of *Bm *YELLOW protein family genes of the silkworm demonstrated are as follows: on one hand, the seven complete members encoding various lengths of amino acids, 284 to 514 long, contained stretches of unique amino acid sequences in their coding regions; on the other hand, some of the 0–12 introns included extensive repeat sequences. But only 1–3 introns were included in the 14 *yellow *family members of *Drosophila melanogaster*. We assumed that the *Bm *YELLOW protein family also originated in the same manner, but the evolutionary procedure was more complex than that in *Drosophila *and *Apis*.

Nothing has been reported on the cloning and function of lepidopteran YELLOW protein family until recently. From the tissue expression profiles, we found that all the seven genes of the *Bm *YELLOW protein family were transcripted in head, wing discs, body wall and gonads (testis and ovary). As *Bm-yellow-d *and *Bm-yellow-b *were observed in every tissue and possessed similar tissue expression patterns, they were also classified into a paraphyletic sub-group in the phylogenetic tree. Then we could speculate that *Bm-yellow-d *and *Bm-yellow-b *had important roles in silkworm development and had similar physiological functions. The *Bm-yellow-fa*, *Bm-yellow-fb *and *Bm-yellow-f2 *had a similar distribution in the tissue expression profiles and had the best similarity to *Drosophila yellow-f *and *yellow-f2 *which had the dopachrome-conversion enzyme activity. In addition, the three members were classified into a paraphyletic sub-group in the phylogenetic tree. These indicated that *Bm-yellow-fa*, *Bm-yellow-fb *and *Bm-yellow-f2 *might have functions in pigmentation. Preliminary experiments showed that appearance of the pupa was dark black, similar to the phenotype of black pupa due to the over expression of *Bm-yellow-d *in pupal stage via a weakened pathogenic baculoviral Expression system (see Additional file 1). This indicated that *Bm-yellow-d *might be in a close relation with cuticle pigmentation too. The function of *Drosophila *YELLOW members is relative to reproduction, and studies showed that *yellow-g *and *yellow-g2 *in *Drosophila *playing a female-specific role in egg development [23]. All the members of the *Bm *YELLOW protein family had a high transcription level in ovary and testis. This suggested that *Bm *YELLOW protein family were also involved to the reproduction.

## Conclusion

*Bombyx mori *YELLOW protein family is the first reported MRJP/YELLOW family to date in the Lepidoptera. It is composed of at least seven members each has one MRJP domain. The low identity of their signal peptides and their MRJP conserved domains, and the highly diversity in cDNA and genomic structure and also unique tissue expression patterns indicated that the members of *Bm *YELLOW protein family might have various functions in the silkworm development.

## Methods

### Insects and tissue dissection

The silkworm stock Jingsong×Haoyue is maintained in our laboratory. The insects were reared on artificial diet at 25°C with 70%–80% relative humidity. Tissues were dissected out at proper developmental stages for the experiments.

### Database searching and sequence assembly

The protein sequence of *Drosophila melanogaster yellow *(GenBank: AAF45497) was used to BLAST *Bombyx mori *EST database [24]. The obtained sequences were associated and catalogued by the use of DNASTAR software package [25] and CLUSTAL X software package [26]. The conserved domain was speculated by the Internet server [27] and the signal peptide was speculated through the SignalP 3.0 Server program [28]. The genomic DNA sequence was searched from the insect genomes with *Bombyx*-limited [29].

### 3'-Rapid amplification of cDNA ends (3'-RACE) and DNA sequencing

Total RNA was isolated from tissues using TRIZOL Reagent (Invitrogen) according to the standard protocol. All RNA samples were treated with Rnase free Dnase (Promega), and evaluated in agarose gels to ensure that they contained intact rRNA and were free of DNA contamination.

One microgramme of total RNA extracted from different tissues at given stages was used for 3'-RACE cDNA synthesis (BD SMART™ RACE cDNA Amplification Kit, Clontech), according to the user manual. PCR was performed with primer1 and Universal Primer A Mix (UPM, Clontech), then a nest PCR was used with primer2 and NUP using the suitable diluted former PCR product as the template. Each PCR reaction was carried out under the following conditions; after denaturing for 5 min at 95°C, subsequent cooling on ice and addition of Taq DNA polymerase, PCR was performed 30 cycles of 94°C for 1 min, 60°C for 1 min, and 72°C for 1–2 min, followed by 10 min incubation at 72°C. Primers used for 3'-RACE were listed in Table 2.

The PCR products were separated on agarose gel by electrophoresis, purified and ligated into pMD18-Tvector (Takara). Several clones were sequenced by the dideoxynucleotide method with ABI-3730 automatic sequencer. DNA sequences were analyzed using DNASTAR and CLUSTAL X software packages.

The *yellow*-like family gene cDNAs obtained in *Bombyx mori *were submitted to the GenBank under the accession numbers listed in Table 1.

### Protein sequences alignment and phylogenetic analysis

Yellow-like protein sequences were aligned and saved as PHYLIP format with CLUSTALX software package. Then a neighbor-joining (NJ) tree based on amino acid sequences was constructed by PHYLIP software package (100 bootstrap replicates).

### RT-PCR

Two microgrammes of total RNA of the different tissues at proper stages were used to synthesize cDNAs according to the Reverse Transcription kit's protocol (Promega Cat.: A3500). Using the reverse transcription products as the templates, 30 cycles of amplification were performed in all the members of *Bm *YELLOW protein family with specific primers listed in Table 2. At the same time, the amplification of *Bm-actin A3 *cDNA (25 cycles) was used as a standardization control. The PCR products were separated on 1.5% agarose gel by electrophoresis.

## Authors' contributions

AHX carried out RT-PCR and data analysis and wrote the manuscript. QXZ carried out tissue dissection, RNA extraction and the cDNA synthesis, participated in the design of the study and helped analyze the data and draft the manuscript. LLY participated in RT-PCR and data analysis. WGL and YZY carried out silkworm breeding and participated in tissue dissection. ZYZ and ZFZ conceived of the study, and participated in its design and coordination and helped to draft the manuscript. All authors read and approved the final manuscript.

## Supplementary Material

Additional File 1 Over expression of *Bm-yellow-d *in pupal stage via a weakened pathogenic baculoviral Expression system. A. Intact one; B. Injected the weak hybridization baculovirus which contained the *beta-galactosidase *gene which is driven by *polyhedrin *promoter as the control; C. Injected a weak hybridization baculovirus which contained the *Bm-yellow-d *gene is driven by *ie-1 *promoter and *hr3 *enhancer expression cassette in the pupa at early stage. From the pictures we could find that the pupa in C is darkened comparing with the controls (A & B). It is known that the *Drosophila yellow *gene is related to normal larval and adult pigmentation, so we thought that the darkness of the pupa in C might be caused by the abundant expression of *Bm-yellow-d*. But we want to declare that the present results are preliminary, and further work is doing in our lab to prove the deduction. Click here for file

## Figures and Tables

**Table 1:** The gene family encoding YELLOW-like proteins in *Bombyx mori*

Name	Related ESTs		Introns	Resource	cDNA length (without Poly(A) tail)	Aa length	MRJP Domain (aa)	Genbank accession no.	Best similar to *Drosophila yellow *family
*Bm-yellow-d*	BP182942	AV404254	6	middle gut/wing discs/ovary/fat body/pheromone gland	1,678	446	122–412	DQ358079	*yellow-d* AAF46993
	BP183166	BP125346							
	BP182340	BP179547							
	BP183224	BP183682							
	BP182019	BP183202							
	AV405837	BP183102							
	BP182816	BP182382							
	CK512445	BP180156							
	CK505477	CK511923							
	BP183653	BP181234							
	BP184192								
									
*Bm-yellow-fa*	CK514230	CK564458	12	embryo/fat body/pheromone gland	1,543	459	168–456	DQ358080	*yellow-f* AAF54884
	BP183402	BP182360							
	BP184163	BP183579							
	CK559440	CK514784							
	BP182690	BP182984							
	CK518098	BP183857							
	CK556095	CK559196							
	BP183954								
									
*Bm-yellow-c*	BP182634	BP182782	>=9	pheromone gland/ovary/fat body	1,916	407	118–406	DQ358081	*yellow-c* AAF54432
	BP184230	BP182899							
	BP183501	BP183088							
	BP179823	CK513620							
	BP182645	BP182022							
									
*Bm-yellow-fb*	CK559703	AV398849	8	ovary/embryo/fat body	1,516	418	132–416	DQ358082	*yellow-f* AAF54884
	CK513741								
									
*Bm-yellow-b*	BP183406		0	pheromone gland	2,045	457	135–413	DQ358083	*yellow-b* AAF59358
									
*Bm-yellow-f2*	BP117152	AU005401	>=5	brain/ovary	1,130	284	125–216	DQ358084	yellow-f2 AAF54885
									
*Bm-yellow*	BP119072	BP117931	>=3	ccompound eye/pheromone gland/ovary/fat body	1,947	514	115–399	DQ358085	*yellow* AAF45497
	BP117822	BP117205							
	BP118364	AU005349							
	BP117579	BP117226							
	BP118757	BP118666							

**Table 2:** Primers used for 3'-RACE and RT-PCR

Primer name	Primers for 3'-RACE	Primers for RT-PCR
Bm-yellow-d	ATCGCTGTTGACAAAGAAACCCTGCA (1) CACGTCCAGTTTTCAACGAGTAATGAC (2)	ATCGCTGTTGACAAAGAAAC (F) GGCGCTGTGTCATTTCTGTAG (R)
Bm-yellow-fa	GAGCGACGGAATATTCAGTCTGACTA (1) TTCCATCCTTTGATCAGCACGCACGA (2)	TTCCATCCTTTGATCAGCACGCACGA (F) TCTAGCATAACCGAAACGCGGCA (R)
Bm-yellow-c	CTGGTCTTGCTGATATTTGGGGTG (1) GAGGACATGAAAGAGGATTCATTCT (2)	ACCTCGAGAGAACGGATTCCGAA (F) CGACCAGGATCCACATTAAGCCT (R)
Bm-yellow-fb	GGATCCGAAGGTATGGGTCAATG (1) CCTACATAACGGACTTGGCGACTA (2)	CCTACATAACGGACTTGGCGACTA (F) AATACACTACTCGGTCCGTGG (R)
Bm-yellow-b	CGATAACGCCATCGGTAACTGCA (1) GATTCTCGATAACGGACGAGTGG (2)	AGGTATTGCATCGATCGCTG (F) GACTAAGCAAGCAGGTGTGCT (R)
Bm-yellow-f2	GGATATCCCAGGTATAAGGAAGC (1) CCGCATTTCAGGAAAGACATCGA (2)	GGATATCCCAGGTATAAGGAAGC (F) GTAATCTGCGCGATTTTGCGCT (R)
Bm-yellow	TCACCTCTGAGCAGTCACACAGAA (1) TCGACCCGTATCTTGAGAGACGAA (2)	TCGACCCGTATCTTGAGAGACGAA (F) TGTGCCTGAAGCACTGGCAATG (R)
Nup and Actin	Nup: AAGCAGTGGTATCAACGCAGAGT	Actin-F: CCCCATCGAACACGGAATCG Actin-R: CGCTCGGCAGTGGTAGTGAA
