# Supplementary material for: Identification and analysis of YELLOW protein family genes in the silkworm, Bombyx mori
Source: BMC Genomics. 2006 Aug 3;7:195. doi: 10.1186/1471-2164-7-195 (PMC1553450; doi:10.1186/1471-2164-7-195)
Supplement: Additional File 1 — Over expression of Bm-yellow-d in pupal stage via a weakened pathogenic baculoviral Expression system. A. Intact one; B. Injected the weak hybridization baculovirus which contained the beta-galactosidase gene which is driven by polyhedrin promoter as the control; C. Injected a weak hybridization baculovirus which contained the Bm-yellow-d gene is driven by ie-1 promoter and hr3 enhancer expression cassette in the pupa at early stage. From the pictures we could find that the pupa in C is darkened comparing with the controls (A & B). It is known that the Drosophila yellow gene is related to normal larval and adult pigmentation, so we thought that the darkness of the pupa in C might be caused by the abundant expression of Bm-yellow-d. But we want to declare that the present results are preliminary, and further work is doing in our lab to prove the deduction. [file 1471-2164-7-195-S1.pdf]

Over expression of *Bm-yellow-d* in pupal stage via a weakened pathogenic baculoviral Expression system

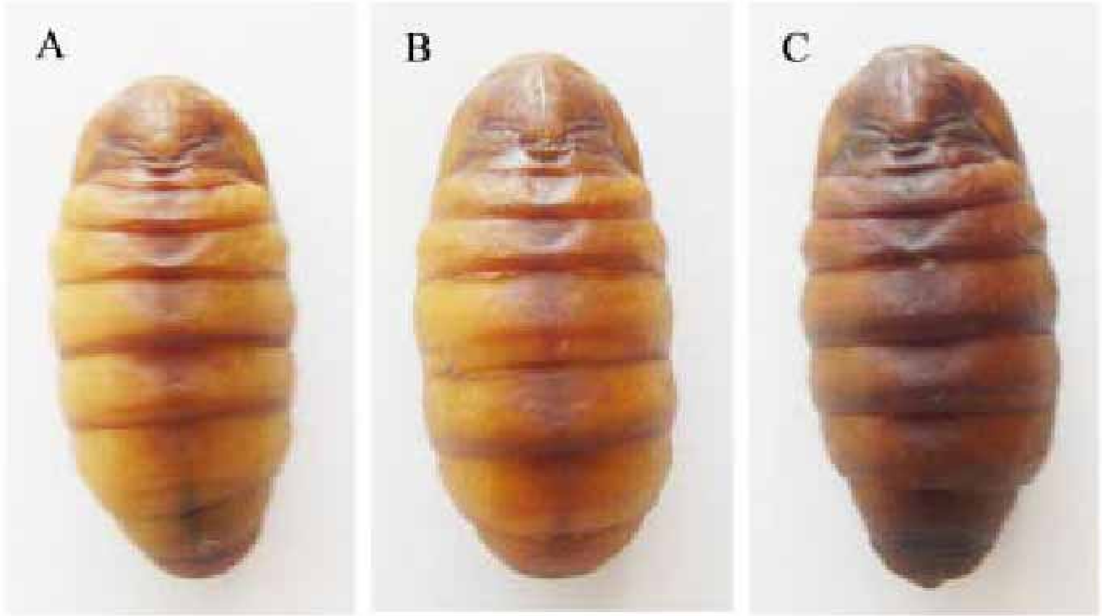

A. Intact one; B. Injected the weak hybridization baculovirus which contained the *beta-galactosidase* gene which is driven by *polyhedrin* promoter as the control; C. Injected a weak hybridization baculovirus which contained the *Bm-yellow-d* gene is driven by *ie-1* promoter and *hr3* enhancer expression cassette in the pupa at early stage. From the pictures we could find that the pupa in C is darkened comparing with the controls (A & B). It is known that the *Drosophila yellow* gene is related to normal larval and adult pigmentation, so we thought that the darkness of the pupa in C might be caused by the abundant expression of *Bm-yellow-d*. But we want to declare that the present results are preliminary, and further work is doing in our lab to prove the deduction.
